# A negative T‐wave in electrocardiogram at 50 years predicted lifetime mortality in a random population‐based cohort

**DOI:** 10.1002/clc.23440

**Published:** 2020-09-10

**Authors:** Helen Sjöland, Michael Fu, Kenneth Caidahl, Per‐Olof Hansson

**Affiliations:** ^1^ Department of Molecular and Clinical Medicine Institute of Medicine, Sahlgrenska Academy, University of Gothenburg Gothenburg Sweden; ^2^ Department of Molecular Medicine and Surgery Karolinska Institutet Stockholm Sweden

**Keywords:** cardiovascular diseases, electrocardiography, longevity, longitudinal studies, mortality, T‐wave inversion

## Abstract

**Background:**

Severe electrocardiographic (ECG) abnormalities in asymptomatic subjects correlate with cardiovascular risk.

**Hypothesis:**

The role of minor ECG abnormalities is less well‐known. We evaluated the association between a negative T‐wave and mortality, as a possible marker for prognosis.

**Methods:**

A prospective, population‐based cohort, examined at 50 years, and followed until death. Time to death (event rates) and predictive role of a negative T‐wave (Cox regression) were analyzed.

**Results:**

Participants (n = 839) with a negative T‐wave (7.3%) had significantly higher blood pressure (BP) (mean systolic 157.9 mmHg vs 136.8 mmHg without negative T‐wave, *P* = <.0001). A negative T‐wave correlated with elevated risk (hazard ratio [HR] [95% CI] [confidence interval]) for all‐cause and cardiovascular (CV) death (1.59 (1.20‐2.11) *P* = .0012 vs 1.91 (1.34‐2.73) *P* = .0004). The association remained after excluding coexisting Q/QS patterns and ST‐junction/segment depression ECG abnormalities (1.66 [1.13‐2.44] *P* = .0098 for all‐cause vs 1.87 [1.13‐3.09] *P* = .015 for CV death). Death from other causes was not associated with a negative T‐wave. A major negative T‐wave carried higher risk than a minor (2.17 [1.25‐3.76] *P* = .0062 vs 1.78 [1.13‐2.79] *P* = .012) for CV death.

**Conclusion:**

A negative T‐wave at 50 years, in asymptomatic individuals, carried an increased risk of all‐cause and CV death during lifetime follow‐up.

## 1 INTRODUCTION

The electrocardiogram (ECG) is a powerful and easily available tool for detection of cardiac disorder in asymptomatic individuals, often preceding symptoms by considerable time.^1^, ^2^, ^3^, ^4^, ^5^ Several studies have linked ECG abnormalities with CV events and prognosis in asymptomatic adults^6^, ^7^, ^8^, ^9^ predominantly for major ECG aberrations.^10^, ^11^, ^12^

Minor ECG abnormalities are common from middle age,^13^, ^14^ and frequently seen in clinical practice in asymptomatic subjects. They are generally regarded as unspecific, not linked to specific myocardial disease.^13^, ^15^, ^16^, ^17^, ^18^ The prognostic value of minor ECG abnormalities is not fully known, but has a potential for preventive considerations. Here, we present a longitudinal population‐based observational cohort study of 50‐year old men, investigating the correlation between a negative T‐wave in resting ECG and mortality during 48 years of follow‐up.

## 2 METHODS

### 2.1 Study population

The “study of men born in 1913” is a longitudinal, prospective, population‐based study, which recruited men born in 1913 from the city of Gothenburg (approximately 500 000 inhabitants at start of study) in western Sweden. From the population registry, a sample was drawn in 1963, consisting of all the men born in 1913 on a day of the month divisible by three (ie, the third, sixth, and ninth day of each month, and so forth). These criteria were fulfilled by 973 men born in 1913, of whom 855 (87.9%) participated in a health examination. Participants and nonparticipants have been previously described.^19^, ^20^ Informed consent was obtained at each examination, orally during the first examinations, as required at the time, and in writing later on, according to the Declaration of Helsinki (established in 1964). Research ethics approval was repeatedly obtained, first from the Research Ethics Committees in Gothenburg and Uppsala, Sweden, and later from the Regional Ethics Review Board, Uppsala, Sweden, No 2011/304.

At baseline, 1963, clinical examination and laboratory analyses were performed. Information on lifestyle, medical history, and smoking habits was collected through a questionnaire. Body weight was measured with a lever balance to the nearest tenth of a kilogram, while body height was measured to the nearest centimeter. Body mass index (BMI) was measured as weight (kg)/height (m^2^). After an overnight fast, blood samples for serum cholesterol, triglycerides, and blood glucose were drawn from an antecubital vein, with light stasis, and analyzed according to standard laboratory procedures.

Blood pressure (BP) Korotkoff phase 1 and 5 were recorded in the right arm, with the participant in the sitting position after a five‐minute rest with a mercury sphygmomanometer cuff size of 12 x 23 cm. Hypertension was defined as the use of antihypertensive medication or current systolic BP ≥140 mmHg and/or a diastolic BP ≥90 mmHg. Diabetes mellitus was defined as self‐reported in the questionnaire. Smoking habits were classified as: 1 = never smoked, 2 = ex‐smoker since 3 months or 3 = current smoker.

Physical activity during leisure time was assessed at a re‐examination in 1967, with the four‐grade Saltin Grimby Physical Activity Scale (SGPALS) and coded as 1 = sedentary, 2 = some light physical activity, such as walking, riding a bicycle, and light gardening for at least 4 hours a week, 3 = regular moderate physical activity for a minimum of 3 hours a week and 4 = regular intense physical training for competition sports.

Standard 12‐lead ECGs were recorded with the patients at rest in the supine position. Paper speed was 50 mm/s and calibration was 1 mV: 10 mm. All of the ECGs were evaluated by a physician, who was blinded to the clinical data, and classified according to the Minnesota Code Classification System for Electrocardiographic Findings.^21^ A negative T‐wave was assessed by the codes 5‐1, 5‐2, 5‐3, and 5‐4 in the classification, where a major negative T‐wave corresponds to codes 5‐1 and 5‐2, and minor negative T‐wave to 5‐3 and 5‐4.^13^, ^22^

### 2.2 Outcome data during lifetime follow‐up

Mortality data were obtained from 1 January 1963 until 1 July 2015 from the National Cause of Death Registry, and included date and cause of death. Cause of death was classified according to the International Classification of Diseases (ICD) codes, ICD 8 until 1986, ICD 9 until 1996, and ICD 10 from 1997 and onwards. Only 14 men (1.6%) were lost to follow‐up over time, primarily due to emigration.

### 2.3 Statistical analysis

All data analyses were performed using SAS software version 9.4 (SAS Institute Inc., Cary, North Carolina). For descriptive purposes, number and percentages were presented for categorical variables and mean, SD, median, minimum, and maximum for continuous variables. For comparison between two groups Fisher's exact test was used for dichotomous variables, Mantel‐Haenszel *χ* ^2^ test for ordered categorical variables and Mann‐Whitney *U* test for continuous variables. Event rates per 100 person years were calculated for time to all‐cause death, death due to CV disease and death due to other causes, with 95% confidence interval (CI) estimated applying exact Poisson limits. Univariable Cox proportional hazards models were used when studying the negative T‐wave's predictive ability on time to death. The proportional hazards assumption was examined by adding an interaction term between negative T‐wave and the logarithm of the follow‐up time and was considered fulfilled. Effect size was described by hazard ratios (HR) and 95% CI. Sensitivity analysis was performed excluding individuals with Q and QS pattern or ST‐junction and segment depression. Interaction analyses between negative T‐wave and baseline clinical characteristics (smoking, physical activity, height, weight, BMI, systolic and diastolic BP, heart rate, BP lowering medication, Q and QS pattern, ST‐junction and segment depression, glucose, cholesterol, hematocrit, and hypertension) were evaluated applying the same statistical methods. These analyses were performed both unadjusted and adjusted for smoking, physical activity, BMI, systolic BP, BP medication, hypertension, glucose, cholesterol, hematocrit, Q and QS pattern, ST‐junction, and segment depression unless the variable is the main variable studied in the interaction. All tests were two‐tailed and conducted at 0.05 significance level.

## 3 RESULTS

### 3.1 Baseline characteristics

At baseline (n = 855), at 50 years, ECG abnormalies were observed in 11.7%, in decreasing frequency: negative T‐wave (7.3%), ST‐junction and segment depression (5.7%), Q/QS patterns (3.0%), and ventricular conduction defects (0.6%). Among these, subjects reporting a prior myocardial infarction (MI) confirmed in medical records (n = 11), or showing ventricular conduction defects (all were left bundle branch block [LBBB]) (n = 5) were excluded (since LBBB includes a negative T‐wave, and was not the subject of analysis). The clinical characteristics for remaining participants (n = 839) are presented relative to a negative T‐wave in Table 1. The findings were balanced between groups for the majority of variables, except for subjects with a negative T‐wave displaying significantly higher prevalence of hypertension (systolic and diastolic BP, use of BP lowering medication, and classified as hypertensive), and lesser height. Coexisting ECG abnormalities were much more frequent in the presence of a negative T‐wave (Table 2), most commonly ST‐junction/segment depression (40.4%). A minor negative T‐wave was overall more common (63.5% of cases) than a major. Among individuals without a negative T‐wave, a majority of 95.4% displayed a completely normal ECG.

**TABLE 1:** Patient characteristics presented by negative T‐wave in 50 year old men born 1913

	Total(n = 839)	Not negative T‐wave(n = 787)	Negative T‐wave(n = 52)	*P* value
Lifestyle				
Smoking				
Nonsmoker	206 (24.6%)	191 (24.3%)	15 (28.8%)	–
Ex‐smoker	163 (19.4%)	151 (19.2%)	12 (23.1%)	–
Current smoker	470 (56.0%)	445 (56.5%)	25 (48.1%)	.28
Physical activity				
Sedentary leisure	287 (35.0%)	272 (35.3%)	15 (30.0%)	–
Moderate exercise during leisure time	265 (32.3%)	244 (31.6%)	21 (42.0%)	–
Regular exercise and training	269 (32.8%)	255 (33.1%)	14 (28.0%)	.99
Missing	18	16	2	–
Anthropometry and BP				
Height (cm)	175.0 (6.0)/175.0 (158.0; 197.0)/ n = 835	175.1 (6.0)/175.0 (158.0; 197.0)/n = 784	173.2 (6.4)/173.0 (160.0; 190.0)/n = 51	.036
Weight (kg)	75.8 (10.9)/75.0 (46.6; 123.5)/n = 835	75.8 (10.7)/75.0 (46.6; 119.0)/n = 784	76.3 (13.0)/75.4 (56.1; 123.5)/n = 51	.88
BMI (kg/m^2^)	24.7 (3.1)/24.6 (16.4; 37.3)/n = 835	24.7 (3.1)/24.5 (16.4; 37.1)/n = 784	25.4 (3.7)/24.9 (18.2; 37.3)/n = 51	.23
Systolic BP (mmHg)	138.1 (20.8)/135.0 (100.0; 240.0)/n = 839	136.8 (19.0)/135.0 (100.0; 225.0)/n = 787	157.9 (34.4)/150.0 (110.0; 240.0)/n = 52	<.0001
Diastolic BP (mmHg)	91.5 (13.2)/90.0 (55.0; 150.0)/n = 839	91.0 (12.5)/90.0 (55.0; 150.0)/n = 787	99.6 (18.9)/95.0 (60.0; 150.0)/n = 52	.0014
Heart rate (bpm)	68.6 (12.0)/67.0 (43.0; 120.0)/n = 839	68.4 (11.9)/67.0 (43.0; 120.0)/n = 787	71.0 (13.2)/70.0 (43.0; 115.0)/n = 52	.079
BP lowering medication	13 (1.5%)	9 (1.1%)	4 (7.7%)	.012
Laboratory data				
Glucose (mmol/L)	4.59 (0.84)/4.55 (2.28; 12.66)/n = 839	4.59 (0.84)/4.55 (2.28; 12.66)/n = 787	4.65 (0.88)/4.61 (3.00; 7.11)/n = 52	.53
Cholesterol (mmol/L)	6.42 (1.11)/6.34 (2.70; 12.53)/n = 839	6.42 (1.10)/6.34 (3.56; 12.53)/n = 787	6.41 (1.28)/6.41 (2.70; 9.57)/n = 52	.96
Hematocrit (%)	44.9 (3.3)/45.0 (32.0; 57.0)/n = 839	44.9 (3.2)/45.0 (32.0; 57.0)/n = 787	45.5 (3.9)/46.0 (33.0; 53.0)/n = 52	.12
Medical history				
Hypertension	567 (67.6%)	524 (66.6%)	43 (82.7%)	.019
Diabetes	11 (1.3%)	10 (1.3%)	1 (1.9%)	1.00

*Note*: For categorical variables n (%) is presented. For continuous variables Mean (SD)/Median (min; max)/n = is presented.

Abbreviations: BMI, body mass index; BP, blood pressure; bpm, beats per minute.

**TABLE 2:** ECG findings by T‐negativity in 1963 in 50 years old men (born 1913 with no previous MI and no LBBB)

ECG variables	Total n (%)	Not negative T‐wave n (%)	Negative T‐wave n (%)	*P* value
Total n	839	787	52	–
Normal ECG	751 (89.5%)	751 (95.4%)	–	–
Negative T‐wave (cat.)				
No negative T‐wave	787 (93.8%)	787 (100.0%)	–	–
Minor negative T‐wave	33 (3.9%)	–	33 (63.5%)	–
Major negative T‐wave	19 (2.3%)	–	19 (36.5%)	–
Q and QS pattern	21 (2.5%)	14 (1.8%)	7 (13.5%)	.0003
ST‐junction and segment depression	44 (5.2%)	23 (2.9%)	21 (40.4%)	<.0001
Q and QS pattern and ST‐junction and segment depression	4 (0.5%)	1 (0.1%)	3 (5.8%)	.0017

*Note*: For categorical variables n (%) is presented.

Abbreviations: BP, blood pressure; ECG, electrocardiogram; LBBB, left bundle branch block; MI, myocardial infarction.

### 3.2 Outcome associated with a negative T‐wave

The mortality associated with a negative T‐wave in ECG was computed (Table 3): For all‐cause death and CV death the event rate was higher in the presence of a negative T‐wave than without. The median follow‐up time was 24.2 vs 29.9 years with vs without a negative T‐wave. A negative T‐wave correlated with elevated risk (HR [95% CI]) for all‐cause and CV death (1.59 [1.20‐2.11] *P* = .0012 vs 1.91 [1.34‐2.73] *P* = .0004). The association remained after excluding coexisting Q/QS patterns and ST‐junction/segment depression ECG abnormalities (1.66 [1.13‐2.44] *P* = .0098 for all‐cause vs 1.87 [1.13‐3.09] *P* = .015 for CV death). A major negative T‐wave carried higher risk than a minor (2.17 [1.25‐3.76] *P* = .0062 vs 1.78 [1.13‐2.79] *P* = .012) for CV death. Death from other causes was not significantly associated with a negative T‐wave.

**TABLE 3:** Mortality (all‐cause, CV and other death) and follow‐up times by T‐wave negativity during the study. Unadjusted Cox proportional hazard models for prediction of time to death, CV death and other death by T‐wave negativity and other concomitant ECG pathology in 50‐year‐old men

		All‐cause death		CV death		Other death	
Predictor	Follow‐up (years)	n (%)	Event rate (95% CI) per 100 person years	n (%)	Event rate (95% CI) per 100 person years	n (%)	Event rate (95% CI) per 100 person years
Not negative T‐wave	29.9 (21.5‐36.1)	777 (98.7%)	3.43 (3.20‐3.68)	406 (51.6%)	1.79 (1.63‐1.97)	371 (47.1%)	1.64 (1.48‐1.81)
Negative T‐wave	24.2 (17.5‐32.9)	52 (100.0%)	4.11 (3.14‐5.40)	33 (63.5%)	2.61 (1.86‐3.67)	19 (36.5%)	1.50 (0.96‐2.36)
	Comparison	Hazard ratio (95% CI)	*P* value	Hazard ratio (95% CI)	*P* value	Hazard ratio (95% CI)	*P* value

Negative T‐wave	Yes vs No	1.59 (1.20–2.11)	.0012	1.91 (1.34‐2.73)	.0004	1.23 (0.78‐1.96)	.37

Negative T‐wave excluding Q and QS pattern and ST‐junction and segment depression	Yes vs No	1.66 (1.13–2.44)	.0098	1.87 (1.13‐3.09)	.015	1.43 (0.78‐2.61)	.24

Negative T‐wave (category)	Minor negative T‐wave vs No negative T‐wave	1.56 (1.10‐2.21)	.013	1.78 (1.13‐2.79)	.012	1.31 (0.75‐2.28)	.34
	Major negative T‐wave vs No negative T‐wave	1.66 (1.05‐2.61)	.030	2.17 (1.25‐3.76)	.0062	1.10 (0.49‐2.46)	.82

Abbreviations: CI, confidence interval; CV, cardiovascular; ECG, electrocardiogram.

### 3.3 Factors that may affect prediction of death by a negative T‐wave

We tested the impact on mortality, all‐cause and CV, of all clinical and demographic variables presented in our models, in the presence of a negative T‐wave, while systematically adjusting for smoking, level of physical activity, BMI, systolic BP, BP medication, hypertension, serum levels of glucose, cholesterol, hematocrit, Q/QS pattern, ST‐junction/segment depression unless the variable was specifically studied in the interaction.

The HR by presence of T‐wave negativity is documented for all variables, for all‐cause and CV mortality (Supplement A and B). Among all tested variables, only three showed significant interaction after adjustment (Table 4): smoking, cholesterol, and hematocrit for all‐cause death. Thus, there was a significantly increased HR for time to all‐cause death for lesser smoking, lower cholesterol levels, and lower hematocrit. For CV death increased risk appeared exclusively for a lower cholesterol level. Notably, for both all‐cause and CV death, the presence of Q/QS pattern and ST‐junction and segment depression lacked significant interaction with the effect of T‐wave negativity (Supplement A and B).

**TABLE 4:** Adjusted Cox proportional hazard models for the adjusted* effect of T‐wave negativity for statistically significant patient characteristics, on time to all‐cause death and CV death

		All‐cause death		CV death	
Predictor	Value	Hazard ratio(95% CI)	*P* value	Hazard ratio(95% CI)	*P* value
Smoking	Nonsmoker	2.41 (1.35‐4.32)	.041	2.39 (1.14‐5.03)	.18
–	Ex‐smoker	0.85 (0.45‐1.61)	–	0.93 (0.41‐2.12)	–
–	Current smoker	1.27 (0.81‐1.99)	–	1.23 (0.69‐2.20)	–
Cholesterol (mmol/L)	5.7 (25th percentile)	2.08 (1.42‐3.05)	.0001	2.00 (1.17‐3.42)	.029
–	7.1 (75th percentile)	1.05 (0.72‐1.52)	–	1.13 (0.71‐1.82)	–
Hematocrit (%)	43 (25th percentile)	1.70 (1.14‐2.53)	.029	1.73 (1.04‐2.89)	.083
–	47 (75th percentile)	1.22 (0.86‐1.73)	–	1.25 (0.80‐1.95)	–

*Note*: *The effect of T‐wave negativity was adjusted for: smoking, physical activity, BMI, systolic BP, BP medication, hypertension, glucose, cholesterol, hematocrit, Q and QS pattern, ST‐junction, and segment depression unless the variable is studied in the interaction.

Abbreviations: BMI, body mass index; BP, blood pressure; CI, confidence interval; CV, cardiovascular.

## 4 DISCUSSION

### 4.1 A negative T‐wave in ECG in middle age is primarily associated with hypertension

In the present study, a negative T‐wave recorded at 50 years predicted all‐cause and CV deaths during lifetime follow‐up in a male random population‐based cohort. We found the presence of hypertension to be the most important differentiating factor between individuals with a negative T‐wave and those without. No significant differences in other CV risk factors were observed. It is well established that ECG abnormalities are more prevalent in hypertension than in normotension and correlates with its severity.^23^ Sustained hypertension may contribute to left ventricular hypertrophy and promote gradually evolving ST‐junction/segment depression and T‐wave items,^2^, ^4^, ^8^ which is linked to adverse CV prognosis.^24^, ^25^, ^26^ However, we consider it noteworthy that hypertension had such considerable association with the presence of a negative T‐wave, already detected at 50 years, and still predictive of lifetime mortality.

We realize, that our baseline findings are representative of a preceding time period, showing ECG abnormalities in 11.7%, and combined ST‐ and T‐wave changes in 8.9% (Table 2) Cohorts from the corresponding era display similar results in middle aged subjects: The Framingham study showed ST‐ and T‐wave changes in 14.1% of 44 to 74 year old males^2^ and the Chicago Western Electric study in 10.3%.^5^ Although cohorts studied at the time differ, ECG abnormalities were generally more prevalent,^2^, ^5^ than in recent observations.^27^ The difference is likely to reflect improved CV health and healthcare at comparable ages over time, also reflected in the remarkably high overall prevalence in hypertension and smoking in our study (Table 1). Similar observations are not expected in the present day, due to improved detection, advances in pharmacologic treatment, and lower cutoff levels for therapy in treatment guidelines.^28^, ^29^

Simultaneous other ECG abnormalities were much more common when a negative T‐wave was present, but limited to ST‐segment/junction depression, most frequently, and also Q/QS pattern. The former would be expected since nondiagnostic ST/T changes are the most common ECG changes overall.^30^ Also conditions causing ECG changes often concomitantly affect ST‐junction/segment levels and T‐wave axis. Thus, we found ST‐segment/junction depression almost 14 times more common when a negative T‐wave was present. Regarding Q/QS pattern, we cannot completely exclude prior silent MI in our cohort, although all confirmed MIs were excluded. Also, a minor negative T‐wave was much more frequent than a major, making up more than 60% of cases, similar to previously reported.^13^

### 4.2 A negative T‐wave as an independent lifetime predictor for risk for death and cardiovascular death

The higher event rate for all‐cause and CV mortality associated with a negative T‐wave was consistent with the presence of more hypertension in this group, and of ECG abnormalities, in general, as markers of compromized CV health.^24^, ^25^, ^26^ The association of a negative T‐wave with CV death, but not non‐CV death, strengthens its role as a lifetime marker for increased CV risk.

Consistent with the above, the HR for death in the presence of a negative T‐wave was also significantly elevated both for all‐cause and CV death, almost doubled for the latter. The increased risk persisted after exclusion of concomitant Q/QS pattern and concomitant ST‐junction/segment depression. This implies that a negative T‐wave marks an elevated risk of death, particularly CV death, and the risk persists, also in the absence of other ECG abnormalities. This is an important finding since the significance of isolated negative T‐waves in an asymptomatic individual has been unclear.

Furthermore, although both major and minor T‐wave inversions carried significantly increased risk for all‐cause and CV death, the risk was most expressed for a major negative T‐wave. Although our focus was on analysis of isolated T‐wave inversion, we propose a similar rationale as for general ECG findings: Major ECG abnormalities are more solidly linked to CV disease and prognosis in asymptomatic individuals, than minor.^1^, ^2^, ^3^, ^4^, ^11^, ^14^ The physiology behind greater aberration from normality is likely to reflect more extensive CV pathology. Therefore, it is reasonable that a major negative T‐wave carries a higher risk than a minor. To our knowledge, there is no prior documented observation of this greater prognostic risk for isolated major T‐wave abnormalities.

Despite adjustment for numerous risk factors, a negative T‐wave remained an independent lifetime predictor for risk for all‐cause and CV death for lower cholesterol levels. For all‐cause death, the increased risk included also lesser smoking and lower hematocrit. All three conditions per se are associated with lower overall CV risk. Our results indicate that a negative T‐wave in absence of other evident CV risk factors may carry a distinctive message of enhanced risk, not to be ignored. When a negative T‐wave is present in middle age, albeit an otherwise favorable risk factor profile, the enhanced risk appears comparatively even larger, and must always be regarded as a marker of CV risk.

However, this study has some limitations to consider. First, the cohort was collected in the 1960s, which makes the results of limited contemporary applicability. Moreover, only men in a limited geographic area were included and the findings cannot be generalized to women and other populations. Second, the size of the cohort was moderate. However, the long‐term follow‐up still gave statistical strength.

## 5 CONCLUSION

A negative T‐wave in ECG registered at 50 years of age in asymptomatic individuals from a randomized observational cohort carried an increased risk of all‐cause and CV death during lifetime follow‐up. The enhanced risk remained after adjustment for other ECG abnormalities and other CV risk factors. A major T‐wave inversion conveyed a higher risk than minor and a negative T‐wave carried enhanced risk also in the absence of other risk factors.

## CONFLICT OF INTEREST

The authors declare no potential conflict of interests.

## Supporting information


**Appendix S1**: Supplement A. Adjusted Cox proportional hazard models for the effect of T‐wave negativity at 50 years for different patient characteristics, on time to all‐cause death.
Supplement B Adjusted Cox proportional hazard models for the effect of T‐wave negativity at 50 years for different patient characteristics, on time to cardiovascular death
