# Supplementary material for: A negative T‐wave in electrocardiogram at 50 years predicted lifetime mortality in a random population‐based cohort
Source: Clin Cardiol. 2020 Sep 10;43(11):1279–85. doi: 10.1002/clc.23440 (PMC7661687; doi:10.1002/clc.23440)
Supplement: Supplementary file 1 — Appendix S1: Supplement A. Adjusted Cox proportional hazard models for the effect of T‐wave negativity at 50 years for different patient characteristics, on time to all‐cause death. Supplement B Adjusted Cox proportional hazard models for the effect of T‐wave negativity at 50 years for different patient characteristics, on time to cardiovascular death [file CLC-43-1279-s001.docx]

**Supplement A.** Adjusted Cox proportional hazard models for the effect of T-wave negativity at 50 years for different patient characteristics, on time to all-cause death.

|  | | Unadjusted | | Adjusted* | |
| --- | --- | --- | --- | --- | --- |
| Predictor | **Value** | **Hazard Ratio (95% CI)** | **P-value** | **Hazard Ratio (95% CI)** | **P-value** |
|  | | | | | |
| Smoking | **Non-smoker** | 2.83 (1.64 - 4.90) | 0.062 | 2.41 (1.35 - 4.32) | 0.041 |
|  | **Ex-smoker** | 1.09 (0.59 - 2.02) |  | 0.85 (0.45 - 1.61) |  |
|  | **Current smoker** | 1.55 (1.03 - 2.32) |  | 1.27 (0.81 - 1.99) |  |
|  | | | | | |
| Physical activity | **Sedentary leisure** | 1.69 (1.00 - 2.85) | 0.93 | 1.32 (0.77 - 2.28) | 0.99 |
|  | **Moderate exercise during leisure time** | 1.59 (1.02 - 2.48) |  | 1.27 (0.76 - 2.11) |  |
|  | **Regular exercise and training** | 1.46 (0.85 - 2.50) |  | 1.34 (0.75 - 2.39) |  |
|  | | | | | |
| Height (cm) | **170** | 1.70 (1.22 - 2.37) | 0.53 | 1.40 (0.96 - 2.05) | 0.50 |
|  | **180** | 1.46 (0.96 - 2.23) |  | 1.19 (0.75 - 1.88) |  |
|  | | | | | |
| Weight (kg) | **67** | 1.64 (1.13 - 2.40) | 0.83 | 1.33 (0.86 - 2.08) | 0.90 |
|  | **84** | 1.56 (1.09 - 2.23) |  | 1.29 (0.88 - 1.91) |  |
|  | | | | | |
| BMI (kg/m^2^) | **22.0** | 1.58 (1.04 - 2.40) | 0.95 | 1.26 (0.78 - 2.05) | 0.84 |
|  | **27.0** | 1.60 (1.15 - 2.23) |  | 1.33 (0.92 - 1.91) |  |
|  | | | | | |
| Systolic blood pressure (mmHg) | **120** | 1.51 (1.00 - 2.28) | 0.85 | 1.36 (0.84 - 2.20) | 0.80 |
|  | **150** | 1.47 (1.10 - 1.97) |  | 1.31 (0.93 - 1.84) |  |
|  | | | | | |
| Diastolic blood pressure (mmHg) | **80** | 1.44 (0.96 - 2.15) | 0.59 | 1.37 (0.86 - 2.19) | 0.91 |
|  | **100** | 1.57 (1.17 - 2.10) |  | 1.40 (1.00 - 1.96) |  |
|  | | | | | |
| Heart rate (bpm) | **59** | 1.67 (1.12 - 2.49) | 0.72 | 1.57 (0.99 - 2.47) | 0.58 |
|  | **77** | 1.53 (1.08 - 2.16) |  | 1.35 (0.92 - 2.00) |  |
|  | | | | | |
| Blood pressure lowering medication | **No** | 1.53 (1.13 - 2.06) | 0.77 | 1.30 (0.92 - 1.83) | 0.78 |
|  | **Yes** | 1.83 (0.56 - 5.94) |  | 1.56 (0.45 - 5.42) |  |
|  | | | | | |
| Q and QS pattern | **No** | 1.57 (1.15 - 2.14) | 0.51 | 1.23 (0.86 - 1.77) | 0.29 |
|  | **Yes** | 2.17 (0.88 - 5.39) |  | 2.10 (0.82 - 5.34) |  |
|  | | | | | |
| ST-junction and segment depression | **No** | 1.68 (1.16 - 2.42) | 0.28 | 1.44 (0.98 - 2.13) | 0.37 |
|  | **Yes** | 1.13 (0.62 - 2.06) |  | 1.03 (0.55 - 1.92) |  |
|  | | | | | |
| Glucose (mmol/L) | **4.0** (25^th^ percentile) | 1.59 (1.13 - 2.24) | 0.98 | 1.31 (0.88 - 1.93) | 1.00 |
|  | **5.1** (75^th^ percentile) | 1.60 (1.16 - 2.20) |  | 1.31 (0.91 - 1.88) |  |
|  | | | | | |
| Cholesterol (mmol/L) | **5.6** (25^th^ percentile) | 2.21 (1.55 - 3.16) | 0.0040 | 2.19 (1.47 - 3.24) | 0.0001 |
|  | **7.3** (75^th^ percentile) | 1.19 (0.83 - 1.70) |  | 0.95 (0.63 - 1.41) |  |
|  | | | | | |
| Hematocrit (%) | **42** (25^th^ percentile) | 1.91 (1.29 - 2.82) | 0.14 | 1.85 (1.19 - 2.87) | 0.029 |
|  | **48** (75^th^ percentile) | 1.36 (0.97 - 1.92) |  | 1.12 (0.77 - 1.64) |  |
|  | | | | | |
| Hypertension | **No** | 1.12 (0.57 - 2.18) | 0.23 | 0.92 (0.44 - 1.92) | 0.28 |
|  | **Yes** | 1.76 (1.28 - 2.42) |  | 1.42 (0.99 - 2.03) |  |
| BMI, body mass index, bpm, beats per minute. * The effect of T-wave negativity adjusted for: smoking, physical activity, BMI, systolic blood pressure, blood pressure medication, hypertension, glucose, cholesterol, hematocrit, Q and QS pattern, ST-junction and segment depression unless the variable is studied in the interaction. | | | | | |

**Supplement B** Adjusted Cox proportional hazard models for the effect of T-wave negativity at 50 years for different patient characteristics, on time to cardiovascular death

|  | | Unadjusted | | Adjusted* | |
| --- | --- | --- | --- | --- | --- |
| Predictor | **Value** | **Hazard Ratio (95% CI)** | **P-value** | **Hazard Ratio (95% CI)** | **P-value** |
|  | | | | | |
| Smoking | **Non-smoker** | 3.30 (1.66 - 6.57) | 0.24 | 2.39 (1.14 - 5.03) | 0.18 |
|  | **Ex-smoker** | 1.49 (0.69 - 3.25) |  | 0.93 (0.41 - 2.12) |  |
|  | **Current smoker** | 1.74 (1.03 - 2.94) |  | 1.23 (0.69 - 2.20) |  |
|  | | | | | |
| Physical activity | **Sedentary leisure** | 1.67 (0.82 - 3.42) | 0.88 | 1.29 (0.61 - 2.71) | 0.93 |
|  | **Moderate exercise during leisure time** | 2.09 (1.20 - 3.63) |  | 1.23 (0.64 - 2.35) |  |
|  | **Regular exercise and training** | 1.78 (0.90 - 3.49) |  | 1.48 (0.71 - 3.08) |  |
|  | | | | | |
| Height (cm) | **170** | 1.99 (1.31 - 3.03) | 0.63 | 1.40 (0.86 - 2.27) | 0.60 |
|  | **180** | 1.72 (0.99 - 2.97) |  | 1.19 (0.67 - 2.14) |  |
|  | | | | | |
| Weight (kg) | **67** | 1.65 (0.98 - 2.78) | 0.44 | 1.22 (0.67 - 2.21) | 0.68 |
|  | **84** | 2.07 (1.36 - 3.15) |  | 1.38 (0.86 - 2.22) |  |
|  | | | | | |
| BMI (kg/m^2^) | **22.0** | 1.47 (0.82 - 2.64) | 0.26 | 1.08 (0.55 - 2.11) | 0.42 |
|  | **27.0** | 2.06 (1.39 - 3.04) |  | 1.40 (0.89 - 2.19) |  |
|  | | | | | |
| Systolic blood pressure (mmHg) | **120** | 1.71 (0.99 - 2.95) | 0.55 | 1.57 (0.83 - 2.99) | 0.44 |
|  | **150** | 1.55 (1.06 - 2.28) |  | 1.37 (0.88 - 2.14) |  |
|  | | | | | |
| Diastolic blood pressure (mmHg) | **80** | 1.75 (1.04 - 2.94) | 0.98 | 1.68 (0.91 - 3.08) | 0.81 |
|  | **100** | 1.74 (1.20 - 2.52) |  | 1.59 (1.04 - 2.45) |  |
|  | | | | | |
| Heart rate (bpm) | **59** | 1.58 (0.92 - 2.70) | 0.32 | 1.31 (0.70 - 2.45) | 0.25 |
|  | **77** | 2.12 (1.40 - 3.22) |  | 1.93 (1.19 - 3.11) |  |
|  | | | | | |
| Blood pressure lowering medication | **No** | 1.84 (1.26 - 2.69) | 0.64 | 1.33 (0.85 - 2.06) | 0.87 |
|  | **Yes** | 1.26 (0.26 - 6.08) |  | 1.16 (0.22 - 6.22) |  |
|  | | | | | |
| Q and QS pattern | **No** | 1.82 (1.22 - 2.72) | 0.64 | 1.20 (0.75 - 1.93) | 0.24 |
|  | **Yes** | 2.40 (0.80 - 7.18) |  | 2.49 (0.80 - 7.72) |  |
|  | | | | | |
| ST-junction and segment depression | **No** | 2.01 (1.27 - 3.20) | 0.38 | 1.37 (0.82 - 2.28) | 0.78 |
|  | **Yes** | 1.33 (0.60 - 2.96) |  | 1.19 (0.51 - 2.76) |  |
|  | | | | | |
| Glucose (mmol/L) | **4.0** (25^th^ percentile) | 1.68 (1.06 - 2.66) | 0.36 | 1.13 (0.67 - 1.93) | 0.28 |
|  | **5.1** (75^th^ percentile) | 2.03 (1.37 - 2.99) |  | 1.45 (0.91 - 2.29) |  |
|  | | | | | |
| Cholesterol (mmol/L) | **5.6** (25^th^ percentile) | 2.47 (1.51 - 4.05) | 0.075 | 2.08 (1.19 - 3.64) | 0.029 |
|  | **7.3** (75^th^ percentile) | 1.47 (0.95 - 2.29) |  | 1.05 (0.63 - 1.74) |  |
|  | | | | | |
| Hematocrit (%) | **42** (25^th^ percentile) | 2.12 (1.28 - 3.52) | 0.45 | 1.88 (1.07 - 3.31) | 0.083 |
|  | **48** (75^th^ percentile) | 1.69 (1.10 - 2.62) |  | 1.15 (0.72 - 1.85) |  |
|  | | | | | |
| Hypertension | **No** | 1.21 (0.50 - 2.97) | 0.28 | 0.99 (0.36 - 2.68) | 0.52 |
|  | **Yes** | 2.10 (1.40 - 3.14) |  | 1.40 (0.88 - 2.22) |  |
| BMI, body mass index, bpm, beats per minute. * The effect of T-wave negativity adjusted for: smoking, physical activity, BMI, systolic blood pressure, blood pressure medication, hypertension, glucose, cholesterol, hematocrit, Q and QS pattern, ST-junction and segment depression unless the variable is studied in the interaction. | | | | | |
